# Why do teachers procrastinate? The role of fear of negative evaluation and intolerance of uncertainty

**DOI:** 10.3389/fpsyg.2026.1881258

**Published:** 2026-08-25

**Authors:** Ema Di Petrillo, Jerónimo Javier Gonález-Bernal, Matteo Villanova, Valeria Caggiano, Juana María Gutiérrez-Caballero, Luna Blázquez-Gutiérrez, Alberto Blázquez-Manzano, Paula Rodríguez-Fernández

**Affiliations:** 1 Business School, Rome, Italy; 2 Department of Health Sciences, University of Burgos, Burgos, Spain; 3 Department of Education, Università degli Studi Roma Tre, Rome, Italy; 4 Department of Psychology and Anthropology, University of Extremadura, Badajoz, Spain; 5 Department of Philosophy, Logic and Aesthetics, University of Salamanca, Salamanca, Spain; 6 Department of Sports, Regional Government of Extremadura, Mérida/Badajoz, Spain; 7 Department of Education and Educational Innovation, Faculty of Law, Education and Humanities, Universidad Europea de Madrid, Madrid, Spain

**Keywords:** academic procrastination, avoidance behavior, fear of negative evaluation, intolerance of uncertainty, teacher wellbeing, teachers

## Abstract

Academic procrastination has increasingly been recognized as a significant source of stress and professional dysfunction among teachers. However, the psychological mechanisms underlying this behavior are not yet fully understood. This study aimed to examine the associations among academic procrastination, intolerance of uncertainty, and fear of negative evaluation in a sample of secondary school teachers, with particular emphasis on the indirect role of fear of negative evaluation in the relationship between intolerance of uncertainty and academic procrastination. A cross-sectional study was conducted with a sample of 444 secondary school teachers who voluntarily participated in the study. Data were collected using the Intolerance of Uncertainty Scale–Short Form (IUS−12), Brief Fear of Negative Evaluation Scale (BFNE), and Academic Procrastination Scale–Short Form (APS–S) were used. Statistical analyses, conducted using SPSS 29.0 and JASP, including descriptive statistics, reliability analyses, Pearson correlations, hierarchical regression, and regression-based mediation analysis with bootstrapping. The results indicated that intolerance of uncertainty was positively associated with academic procrastination (B = 0.088, SE = 0.030, *p* = 0.004). However, this association became non-significant when fear of negative evaluation was included in the model (B = 0.026, SE = 0.031, *p* = 0.401). In addition, a significant indirect association between intolerance of uncertainty and academic procrastination through fear of negative evaluation was observed [indirect effect = 0.064, 95% CI (0.036, 0.095)]. Academic procrastination appears to be closely linked to fear of negative evaluation in contexts characterized by uncertainty among secondary school teachers. These findings support the relevance of fear of negative evaluation as a key mechanism associated with procrastination and highlight the importance of targeting these processes in interventions aimed at reducing avoidance-related behaviors and promoting teachers' wellbeing.

## 1 Introduction

Procrastination is a complex and poorly understood behavior that involves various cognitive, emotional, and behavioral components (Burka and Yuen, 1983; Díaz-Morales, 2019; Steel, 2007). It has been viewed as a failure of self-regulation and has consistently been associated with low self-efficacy (Klassen et al., 2008).

More specifically, academic procrastination has been extensively studied in students (Aldalhalm, 2022; Bera and Gadatia, 2021; García-Ros et al., 2017; Shaked and Altarac, 2022), where it has been defined as the tendency to voluntarily and unnecessarily delay engagement in academic tasks and activities despite the harmful effects that failing to complete them may entail (Irfan et al., 2024).

Its consequences have been extensively reviewed in this context, being associated with low academic performance, difficulties in self-regulation, and negative impacts on psychological wellbeing (Ramadhani et al., 2026; Solomon and Rothblum, 1984; Steel, 2007; Ahmed et al., 2023). However, its analysis among teachers or future teachers has been less explored (Enríquez Félix, 2026; Irfan et al., 2024).

Arulselvi and Singaravelu (2019) explored the relationship between academic procrastination and the pedagogical competence of pre-service teachers and found that more than half exhibited a moderate level of academic procrastination. Teachers' procrastination can manifest itself in delays in tasks such as lesson planning, grading assessments, completing administrative paperwork, or preparing educational activities, with direct implications for professional wellbeing and the quality of the educational process (Enríquez Félix, 2026). These professional tasks share key characteristics with academic activities, supporting the extension of the concept of academic procrastination to teaching contexts and highlighting the relevance of examining this phenomenon in practicing teachers.

Beyond interpretations focused on deficits in self-regulation or time management problems, recent research has proposed conceptualizing procrastination as a form of emotional avoidance (González-Brignardello et al., 2023). Thus, postponing tasks allows for a temporary reduction in the distress associated with activities perceived as aversive, complex, or evaluative. The proposed conceptualization is particularly relevant in the teaching profession, where professional performance takes place in a context characterized by uncertainty, ambiguous outcomes, and constant exposure to evaluation by multiple stakeholders, such as students, families, school administrators, or educational authorities (Cárdenas Rodríguez et al., 2014).

This approach is grounded in transdiagnostic models of emotional vulnerability, which argue that certain emotional vulnerabilities, such as intolerance of uncertainty, increase the likelihood that, in specific contexts, more specific cognitive-emotional mechanisms will be triggered, leading to avoidance behaviors (Carleton, 2012; Rosser, 2019). Similarly, several studies have linked intolerance of uncertainty to anxiety, excessive worry, and avoidant behaviors, as well as to difficulties in initiating or maintaining actions in unstructured contexts (McEvoy et al., 2019). From this perspective, intolerance of uncertainty may contribute to procrastination by increasing the perceived ambiguity and aversiveness of tasks, leading individuals to delay task initiation to regulate distress. In teaching contexts, where tasks often involve uncertain outcomes and evaluative components, this dispositional tendency may intensify avoidance behaviors (Carleton, 2012).

However, the relationship between uncertainty intolerance and procrastination is not necessarily direct. Many theoretical models propose that the effects of this dispositional vulnerability do not manifest directly but are channeled through specific cognitive-emotional mechanisms (Rosser, 2019; Li et al., 2020). From this perspective, fear of negative evaluation has been identified as a mechanism underlying the relationship between intolerance of uncertainty and maladaptive emotional responses (Spiroiu and Maranzan, 2025). This construct, understood as a persistent concern about being judged unfavorably, has been shown to mediate the relationship between emotional vulnerability and distress in evaluative situations (Sigurvinsdottir et al., 2021).

In this sense, people with high intolerance of uncertainty may exhibit increased sensitivity to ambiguous evaluative contexts, which heightens concerns about negative judgment and potential failure. In settings such as education, where uncertainty and evaluation are pervasive, these concerns can intensify the perception of tasks as threatening, increasing negative emotional arousal and fostering avoidant responses (Morriss et al., 2023). From this perspective, procrastination can be understood as an avoidance response to the anticipation of negative evaluation, rather than as a direct consequence of uncertainty itself. Consequently, this behavior may function as a strategy aimed at reducing the emotional distress associated with such evaluative expectations. However, although there is evidence linking anxiety, fear of negative evaluation, and procrastination (Hameed et al., 2025), studies that explicitly integrate intolerance of uncertainty, fear of negative evaluation, and procrastination into a single explanatory model remain limited.

The present study addresses this gap by examining these relationships in a sample of practicing secondary school teachers, providing insight into how these processes operate in professional contexts.

Specifically, the study aims to examine the associations among intolerance of uncertainty, fear of negative evaluation, and academic procrastination, with particular emphasis on the indirect role of fear of negative evaluation in the relationship between intolerance of uncertainty and academic procrastination. Accordingly, it is hypothesized that fear of negative evaluation will be positively associated with academic procrastination, representing a proximal factor of this behavior, whereas intolerance of uncertainty will also be positively related as a more distal vulnerability factor. Furthermore, fear of negative evaluation is expected to be indirectly associated with the relationship between intolerance of uncertainty and academic procrastination.

## 2 Materials and methods

### 2.1 Study design and procedure

A quantitative, non-experimental study was conducted using a cross-sectional design with an explanatory correlational scope. This design allowed us to analyze the relationships between psychological variables associated with academic procrastination among secondary school teachers, as well as to evaluate an explanatory model based on indirect relationships among these variables.

Data were collected using a self-administered online questionnaire, which was disseminated via the professional social network LinkedIn and aimed at active Spanish secondary school teachers. The questionnaire was administered at a single point in time, from April to June 2025, and the estimated completion time was approximately 15 min.

Before accessing the questionnaire, participants received detailed information about the study's objectives, the voluntary nature of their participation, and the guarantee of anonymity and confidentiality of their responses. Informed consent was explicitly obtained prior to the start of the questionnaire.

### 2.2 Participants

Convenience sampling was used to select the participants. A total of 600 secondary school teachers were invited to participate in the study, of whom 444 completed the questionnaire (response rate = 74.0%). Participants were recruited through educational institutions and professional networks, and participation was voluntary and anonymous. Inclusion criteria required participants to be active secondary school teachers working in Spain. No specific exclusion criteria were established beyond not meeting these conditions.

The use of convenience sampling was justified by the exploratory nature of the study and the difficulty of accessing a fully representative sample of teachers across different educational contexts. Regarding sample size, no a priori power analysis was conducted. However, the final sample size (*N* = 444) was considered adequate to detect small-to-moderate effect sizes in multiple regression analyses, ensuring sufficient statistical power for the analyses performed.

The participants' ages ranged from 23 to 65 years (M = 41.76; SD = 10.80), and their teaching experience in secondary education ranged from 0 to 39 years (M = 11.17; SD = 9.95). The sample was evenly distributed by gender (50% men and 50% women).

Regarding other sociodemographic and professional characteristics, most participants were married or in a stable relationship, approximately half had children, and the majority were tenured or temporary teachers. Most teachers worked in schools located in urban settings. The detailed characteristics of the sample are presented in Tables 1 and 2.

**Table 1:** Sociodemographic characteristics of the sample. Categorical variables.

Variable	*n*	%
**Gender**		
Male	222	50.0
Female	222	50.0
**Marital status**		
Single	123	27.7
Married/in a relationship	293	66.0
Separated/divorced	28	6.3
**Children**		
No children	218	49.1
With children	226	50.9
**Employment status**		
Civil servant	189	42.6
Interim teacher	120	27.0
Permanent contract	113	25.5
Temporary contract	14	3.2
Unemployed	7	1.6
Self-employed	1	0.2
**Professional role**		
Teacher	263	59.2
Coordination/department head	141	31.8
Academic leadership	19	4.3
School management	18	4.1

**Table 2:** Sociodemographic characteristics of the sample. Continuous variables.

Variable	M	SD	Range
Age (years)	41.76	10.80	23–65
Years of teaching experience	11.17	9.95	0–39
Number of children	1.00	1.23	0–10

### 2.3 Data collection tools

An *ad hoc* questionnaire was designed to collect sociodemographic information, including variables such as age, gender, teaching experience, and other relevant professional characteristics. In addition, validated standardized instruments were used to assess the main study variables: Intolerance of Uncertainty Scale–Short Form (IUS−12), Brief Fear of Negative Evaluation Scale (BFNE), and Academic Procrastination Scale–Short Form (APS–S).

#### 2.3.1 Ad hoc questionnaire

Sociodemographic and professional variables were collected to allow for sample characterization and adjustment for potential confounding factors in the statistical analyses.

First, age, measured in full years, and years of teaching experience in secondary education were recorded; these are continuous variables that allow for the exploration of potential associations with teachers' life and professional trajectories. Both variables were subsequently used as covariates in the regression and mediation analyses, given that the prior literature suggests that the management of emotional distress and evaluative demands may vary depending on age and professional experience. Gender was coded dichotomously and used exclusively for descriptive and control purposes, not as part of the explanatory model.

As part of the sociodemographic profile, indicators related to family status were also included to describe the participants' personal circumstances. These variables were used just for descriptive purposes.

Concerning the professional context, employment status (e.g., civil servant, interim, or temporary contract), the role held at the school (exclusively teaching or other coordination or management positions) and the type of school environment based on the town's population were recorded. These variables provided a detailed description of the sample's professional profile but were not directly incorporated into the main analytical models.

#### 2.3.2 Intolerance of Uncertainty Scale–Short Form (IUS 12)

Intolerance of uncertainty was assessed using the IUS−12 (Carleton et al., 2007), in its validated Spanish version (Núñez Parra, 2019). The scale consists of 12 items rated on a five-point Likert scale ranging from 1 (not at all characteristic of me) to 5 (entirely characteristic of me). Total scores are obtained by summing the items, with higher scores indicating greater levels of intolerance of uncertainty.

Furthermore, the scale provides information regarding two distinct dimensions: Prospective Intolerance of Uncertainty (IUP), which refers to anxiety about the future and the tendency to seek information in order to reduce uncertainty; and Inhibitory Intolerance of Uncertainty (IUI), which reflects behavioral inhibition or paralysis in response to uncertain situations. Subscale scores are computed by summing the corresponding items, with higher values reflecting greater levels of each dimension.

Spanish validation studies have demonstrated adequate psychometric properties of the instrument. Regarding reliability, the scale showed adequate internal consistency, with Cronbach's alpha coefficients around 0.85 for the total score, and good test–retest stability (Núñez Parra, 2019).

#### 2.3.3 Brief Fear of Negative Evaluation Scale (BFNE)

Fear of negative evaluation was assessed using the BFNE, originally developed by Leary (1983) based on the Fear of Negative Evaluation Scale by (Leary, 1983), using its Spanish validated version. The scale consists of 12 items with a five-point Likert scale ranging from 1 (not at all characteristic of me) to 5 (extremely characteristic of me). Total scores are obtained by summing the items, with higher scores indicating a greater fear of negative evaluation.

Of the 12 items, four are reverse scored; these were recoded prior to computing the total score.

Validation studies in the Spanish population have demonstrated adequate psychometric properties of the instrument. In terms of reliability, high internal consistency values have been reported, with Cronbach's alpha coefficients exceeding 0.88, as well as adequate composite reliability coefficients and evidence of convergent validity with other measures of social anxiety (Gallego et al., 2007; Gallego, 2010).

#### 2.3.4 Academic Procrastination Scale–Short Form (APS-S)

Academic procrastination was assessed using the Spanish version of the APS–S (Alegre et al., 2022). The APS–S was originally developed by Yockey (2016) and consists of five items rated on a five-point Likert scale ranging from 1 (never) to 5 (always). Total scores are obtained by summing the items, with higher scores indicating a greater tendency toward academic procrastination. The last item is reverse scored and was recoded prior to calculating the total score.

The Spanish version of the APS–S has demonstrated adequate psychometric properties. In adaptation and validation studies among a Spanish speaking population, confirmatory factor analyses confirmed the instrument's unifactorial structure, with excellent fit indices and high levels of reliability such as Cronbach's alpha coefficients above 0.87.

In the context of the present study, academic procrastination was conceptualized as delays in work-related tasks that are inherent to the teaching profession. These include activities such as lesson planning, preparation of instructional materials, grading assessments, administrative duties, and engagement in professional development activities. Although the APS-S was originally developed for student populations, its use was considered appropriate in this context given the academic nature of many teaching-related tasks.

### 2.4 Data analysis

Statistical analyses were performed using IBM SPSS Statistics version 29.0 and JASP. First, descriptive statistics were calculated, and the internal reliability of the instruments was examined using Cronbach's alpha. Then, Pearson's correlation analyses were conducted to examine the bivariate relationships among the main variables.

Preliminary analyses were conducted using sociodemographic variables (age, gender, and years of teaching experience) to identify potential control variables. Subsequently, a hierarchical linear regression analysis was performed to examine the associations between intolerance of uncertainty and fear of negative evaluation on academic procrastination. In the first block, sociodemographic variables were included; in the second block, uncertainty intolerance was added; and in the third block, fear of negative evaluation was incorporated.

Finally, the indirect association between intolerance of uncertainty and academic procrastination through fear of negative evaluation was examined using a regression-based mediation approach with bootstrapping. The analysis was conducted using 5,000 bootstrap resamples, controlling for sociodemographic covariates. Statistical mediation was considered supported when the indirect effect was significant, as indicated by a 95% confidence interval that did not include zero.

In addition, although the present study did not aim to validate the instruments, preliminary confirmatory factor analyses (CFA) were conducted to examine the internal structure of the scales in the current sample. Given the ordinal nature of the items, the Diagonally Weighted Least Squares (DWLS) estimator was used. Model fit was evaluated using the root mean square error of approximation (RMSEA) and the standardized root mean square residual (SRMR), considering values ≤ 0.08 as indicative of acceptable fit. When necessary, theoretically justified modifications were explored, including the modeling of method effects associated with reverse-coded items and the inclusion of residual correlations between items with similar content, following a parsimonious approach.

The level of statistical significance was set at a significance level of *p* < 0.05.

### 2.5 Ethical approval

The research plan was submitted to the Research Ethics Committee of the University of Burgos, and the required authorization was obtained under registration number 032.2/2021. Additionally, informed consent was obtained from all participants prior to their inclusion in the study, confirming their voluntary participation and their agreement that the data collected would be used exclusively for scientific purposes. Participants were clearly informed of their right to withdraw from the study at any point during the research process without penalty or negative consequences. All data processing procedures complied with the European General Data Protection Regulation (GDPR) and with Organic Law 3/2018 on the Protection of Personal Data and Guarantee of Digital Rights.

All procedures carried out within the scope of this study were conducted in accordance with the ethical principles set forth in the Declaration of Helsinki (1964) and its subsequent revisions.

## 3 Results

### 3.1 Sample characteristics

The sample consisted of 444 secondary school teachers. Participants ranged in age from 23 to 65 years, with a mean age of 41.76 ± 10.80). Teaching experience in secondary education ranged from 0 to 39 years, with a mean age of 11.17 ± 9.95. Approximately half of the participants reported having children (50.9%), with a mean of 1.00 ± 1.23 children.

The distribution by sex was balanced, with 50.0% of participants identifying as male and 50.0% as female. Regarding marital status, most participants were married or in a stable relationship (66.0%), followed by those who were single (27.7%) and those separated or divorced (6.3%).

In terms of employment status, most participants were civil servants (42.6%) or interim teachers (27.0%), followed by teachers with permanent contracts (25.5%). Smaller proportions reported temporary contracts, unemployment, or self-employment. With respect to professional roles, most participants worked exclusively as classroom teachers (59.2%), whereas others held coordination or leadership positions. Finally, most teachers reported working in urban contexts, with 58.3% indicating that their workplace was in cities with more than 50,000 inhabitants. Descriptive statistics for sociodemographic variables are presented in Tables 1 and 2.

### 3.2 Internal structure of the measures

To ensure the adequate functioning of the instruments in the present sample, confirmatory factor analyses (CFA) were conducted for each scale.

For the BFNE, the initial unidimensional model showed inadequate fit. Given the known influence of reverse-coded items, a method factor was incorporated to account for the shared variance associated with these items. The resulting model showed an excellent fit [CFI = 1.000, TLI = 1.000, RMSEA = 0.069, 90% CI (0.049, 0.089); SRMR = 0.045], supporting the unidimensional structure of the scale once method effects were controlled.

For the IUS-12, a two-factor structure was estimated. The final model showed a good overall fit according to incremental fit indices (CFI = 0.991, TLI = 0.988; SRMR = 0.055), although the RMSEA value [RMSEA = 0.088, 90% CI (0.077, 0.100)] indicated a marginal level of fit. This result suggests some degree of model complexity, potentially related to item-level dependencies, but was considered acceptable given the theoretical structure of the scale and the modifications applied.

Finally, the APS-S showed an excellent unidimensional fit [CFI = 1.000, TLI = 1.000, RMSEA = 0.076, 90% CI (0.040, 0.116); SRMR = 0.017]. All items showed adequate levels of explained variance after correct recoding of the reverse-scored item.

Overall, these results support the adequate internal structure and functioning of the instruments in the present sample and justify their use in subsequent analyses.

### 3.3 Descriptive statistics and reliability of the measures

Descriptive statistics and internal consistency coefficients for the study variables are presented in Table 3. All instruments demonstrated adequate to excellent reliability in the present sample. Cronbach's alpha coefficients were 0.912 for the BFNE, 0.801 for the APS-S, and 0.880 for the IUS-12. The subscales of the IUS-12 also showed good internal consistency, with alpha coefficients of 0.830 for IUP and 0.856 for IUI.

**Table 3:** Descriptive statistics and reliability of the study variables.

Variable	M	SD	Range	Cronbach's α
IUS total	32.48	8.68	12–60	0.880
IUS-IUP	21.49	5.39	7–35	0.830
IUS-IUI	10.99	4.44	5–25	0.856
BFNE	33.37	10.71	8–60	0.912
APS-S	11.67	5.50	4–25	0.801

These results are consistent with previous validation studies conducted in Spanish populations, which have reported adequate reliability and validity for these instruments.

Mean scores indicated moderate levels of intolerance of uncertainty, fear of negative evaluation, and academic procrastination within the sample.

### 3.4 Correlational analyses

Pearson correlation analyses were conducted to examine the relationships among intolerance of uncertainty, fear of negative evaluation, and academic procrastination. As shown in Table 4, intolerance of uncertainty was positively and significantly associated with academic procrastination (*r* = 0.159, *p* < 0.001). Intolerance of uncertainty was also strongly related to fear of negative evaluation (*r* = 0.393, *p* < 0.001), which in turn showed a moderate positive association with academic procrastination (*r* = 0.300, *p* < 0.001).

**Table 4:** Pearson correlations among study variables.

Variable	IUS total	APS-S	BFNE	IUI	IUP
IUS total	—	0.159^**^	0.393^**^	0.127^*^	0.265^**^
APS-S	0.159^**^	—	0.300^**^	0.038	0.265^**^
BFNE	0.393^**^	0.300^**^	—	0.105^*^	0.281^**^
IUI	0.127^*^	0.038	0.105^*^	—	0.412^**^
IUP	0.265^**^	0.265^**^	0.281^**^	0.412^**^	—

^**^p < 0.01; ^*^p < 0.05.

Regarding the dimensions of intolerance of uncertainty, inhibitory intolerance of uncertainty was not significantly associated with academic procrastination, whereas prospective intolerance of uncertainty showed a moderate positive correlation with procrastination (*r* = 0.265, *p* < 0.001). Both dimensions were positively associated with fear of negative evaluation, with particularly strong associations observed for prospective intolerance of uncertainty.

### 3.5 Preliminary analyses with sociodemographic variables

Additional preliminary analyses examined the relationships between sociodemographic variables and the main study constructs. Age was negatively associated with academic procrastination (*r* = −0.172, *p* < 0.001), fear of negative evaluation (*r* = −0.215, *p* < 0.001), and intolerance of uncertainty (*r* = −0.197, *p* < 0.001). Teaching experience showed negative associations with intolerance of uncertainty (*r* = −0.178, *p* < 0.001) and fear of negative evaluation (*r* = −0.182, *p* < 0.001) but was not significantly related to academic procrastination (*r* = −0.079, *p* = 0.097). A strong positive association was observed between age and teaching experience (*r* = 0.777, *p* < 0.001).

Independent samples *t-*tests indicated no significant differences in academic procrastination by sex [*t*_(442)_ = 1.00, *p* = 0.318].

Finally, age, sex, and teaching experience were included as covariates in subsequent regression and mediation analyses.

Given the high correlation between age and teaching experience, multicollinearity diagnostics were conducted. The results indicated acceptable levels (VIF values ranged from 2.12 to 3.35; tolerance values ranged from 0.45 to 0.70), suggesting that multicollinearity did not pose a significant concern in the present analyses. Therefore, both variables were retained in the models given their theoretical relevance.

### 3.6 Hierarchical regression analysis

A hierarchical multiple regression analysis was conducted to examine whether intolerance of uncertainty and fear of negative evaluation were associated with academic procrastination, controlling for sociodemographic variables (age, sex, and teaching experience).

In Model 1, sociodemographic variables were entered and accounted for a small but statistically significant proportion of variance in academic procrastination (*R*^2^ = 0.039, *p* < 0.001). Age was negatively associated with procrastination (*p* < 0.001), whereas sex and teaching experience were not statistically significant predictors.

In Model 2, intolerance of uncertainty was added, resulting in a significant increase in explained variance (ΔR^2^ = 0.018, *p* = 0.004). Intolerance of uncertainty was positively associated with academic procrastination (*p* = 0.004), beyond the effects of sociodemographic variables.

Finally, in Model 3, fear of negative evaluation was included, producing a further significant increase in explained variance (Δ*R*^2^ = 0.062, *p* < 0.001), with the final model explaining 11.9% of the variance (*R*^2^ = 0.119). Fear of negative evaluation emerged as a significant positive predictor of academic procrastination (*p* < 0.001). In contrast, intolerance of uncertainty was no longer significantly associated with procrastination.

Overall, these results indicate that the association between intolerance of uncertainty and academic procrastination is attenuated when fear of negative evaluation is included in the model. Detailed results are presented in Table 5.

**Table 5:** Hierarchical regression analysis predicting academic procrastination.

Predictor	Model 1			Model 2			Model 3		
	B	SE	β	B	SE	β	B	SE	β
Age	−0.143	0.038	−0.280^***^	−0.133	0.038	−0.261^***^	−0.113	0.037	−0.221^**^
Sex	−0.425	0.517	−0.039	−0.556	0.515	−0.051	−0.939	0.503	−0.085
Teaching experience	0.079	0.041	0.143	0.086	0.041	0.155^*^	0.088	0.040	0.159^*^
Intolerance of uncertainty	—	—	—	0.088	0.030	0.139^**^	0.026	0.031	0.041
Fear of negative evaluation	—	—	—	—	—	—	0.142	0.026	0.276^***^
*R^2^*	0.039			0.057			0.119		
Δ*R^2^*	—			0.018			0.062		

B, unstandardized coefficients; SE, standard error; β, standardized coefficient.

^***^p < 0.001; ^**^p < 0.01; ^*^p < 0.05.

### 3.7 Mediation analysis

A mediation analysis was conducted using a bootstrapping procedure with 5,000 resamples to examine whether fear of negative evaluation statistically mediated the relationship between intolerance of uncertainty and academic procrastination, controlling for age, sex, and teaching experience.

The total association between intolerance of uncertainty and academic procrastination was significant (B = 0.088, SE = 0.030, *p* = 0.004). Intolerance of uncertainty was also significantly associated with fear of negative evaluation (B = 0.452, SE = 0.045, *p* < 0.001), which, in turn, was positively associated with academic procrastination when controlling for intolerance of uncertainty (B = 0.142, SE = 0.026, *p* < 0.001).

The indirect association between intolerance of uncertainty and academic procrastination through fear of negative evaluation was significant [indirect effect = 0.064, SE = 0.015, 95% CI (0.036, 0.095)], as the confidence interval did not include zero.

When fear of negative evaluation was included in the model, the direct association between intolerance of uncertainty and academic procrastination was no longer statistically significant (B = 0.026, SE = 0.031, *p* = 0.401), suggesting that fear of negative evaluation may be associated with the relationship between intolerance of uncertainty and academic procrastination at a statistical level. The magnitude and confidence interval of the indirect association provide the primary basis for interpreting the observed pattern. Overall, these findings support a mediation model characterized by an indirect association between intolerance of uncertainty and academic procrastination through fear of negative evaluation.

**Figure 1:**
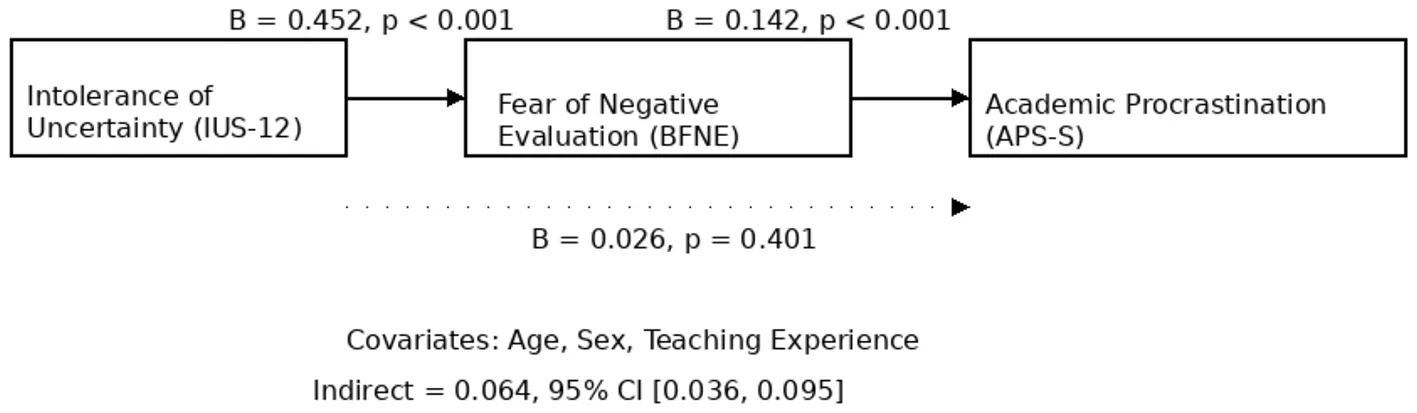
Mediation model of the association between intolerance of uncertainty and academic procrastination through fear of negative evaluation.

This model is graphically represented in Figure 1, which illustrates the associations among intolerance of uncertainty, fear of negative evaluation, and academic procrastination. The figure also displays the non-significant direct association between intolerance of uncertainty and academic procrastination when fear of negative evaluation is included, as well as the significant indirect association obtained through bootstrapping.

## 4 Discussion

The aim of this study was to examine the relationship between academic procrastination and intolerance of uncertainty among secondary school teachers, as well as the mediating role of fear of negative evaluation in that relationship. The results provide consistent evidence that the relationship between intolerance of uncertainty and procrastination is not direct, but is indirectly associated through fear of negative evaluation, supporting a statistical mediation model. These findings contribute to a more accurate understanding of teacher procrastination as an avoidance-related behavior associated with emotional processes, rather than merely an organizational or volitional problem. A comparable pattern was observed by Spiroiu and Maranzan (2025), who demonstrated that fear of negative evaluation may be associated with the relationship between intolerance of uncertainty and maladaptive responses, such as procrastination, even outside the educational context. In line with current methodological recommendations, the interpretation of these findings focuses on the magnitude, statistical significance, and confidence interval of the indirect association rather than on the classification of mediation based on the direct association.

In the present study, the findings indicate that intolerance of uncertainty is significantly associated with academic procrastination, consistent with previous research linking this construct to avoidant behaviors in contexts of ambiguity or unpredictability (McEvoy et al., 2019). Teaching, especially in secondary education, involves high exposure to uncertain situations, such as external assessments, performance observations, constant interaction with students and families, and changing administrative demands. In this context, lower tolerance for uncertainty can generate intense distress when faced with open-ended professional tasks or those with outcomes that are difficult to control, favoring procrastination as a short-term emotional regulation strategy (Mohammadi Bytamar et al., 2020; Morriss et al., 2023).

However, it is important to note that the magnitude of the observed associations was relatively small. For example, the correlation between intolerance of uncertainty and academic procrastination was modest, and the final regression model explained a limited proportion of the variance in procrastination. This indicates that, although the identified relationships are statistically significant, a substantial part of the variability in academic procrastination remains unexplained. Therefore, these findings should be interpreted with caution and understood as contributing to a broader set of factors involved in procrastination rather than providing a comprehensive explanatory model. These results suggest that additional psychological, contextual, and organizational variables may play an important role in explaining procrastination among teachers.

Against this background, the central finding of the study is that this relationship is attenuated and becomes non-significant when fear of negative evaluation is included in the model. Specifically, fear of negative evaluation emerged as a robust predictor of academic procrastination and may be associated with the relationship at a statistical level between intolerance of uncertainty and procrastination. This pattern is consistent with the mediation analysis, which showed a significant indirect association between intolerance of uncertainty and procrastination through fear of negative evaluation, while the direct association was no longer significant. From this perspective, procrastination may be associated with avoidance-related responses to evaluative concerns activated in uncertain contexts, rather than as a direct consequence of intolerance of uncertainty itself. These findings are consistent with transdiagnostic models, which propose that broad dispositional vulnerabilities, such as intolerance of uncertainty, operate through more specific cognitive-emotional mechanisms depending on contextual demands (Carleton, 2012; Rosser, 2019).

In line with previous research (Spiroiu and Maranzan, 2025), the present findings suggest that fear of negative evaluation may be associated with the relationship between intolerance of uncertainty and maladaptive responses. In educational settings, where evaluation is a pervasive and often unavoidable component of professional performance, concerns about being judged negatively may become particularly salient and be linked to avoidance behaviors such as procrastination. It is important to note that, in the present study, academic procrastination refers to delays in teaching-related tasks with an academic nature. This conceptualization supports the use of the APS-S in a sample of practicing teachers, as many professional activities in teaching involve characteristics similar to academic tasks, such as lesson planning, evaluation, and continuous learning processes.

Additionally, correlational analyses showed that the dimensions of intolerance of uncertainty are not equally related to procrastination. Specifically, prospective intolerance of uncertainty exhibited more consistent associations with academic procrastination than inhibitory intolerance. This pattern suggests that cognitive anticipation of future negative outcomes, rather than immediate behavioral inhibition, may be particularly relevant in explaining task delay. This interpretation reinforces the view of procrastination as a form of anticipatory avoidance in response to perceived evaluative threats (González-Brignardello et al., 2023; Carleton, 2012; Rosser, 2019).

Regarding sociodemographic variables, the results indicate that age is negatively associated with procrastination, while years of teaching experience do not show a direct relationship with this behavior. This pattern suggests that certain age-related maturation or emotional regulation processes may contribute to better management of evaluative distress, regardless of accumulated professional experience. In contrast to our results, Enríquez Félix (2026) found a negative correlation between years of teaching experience and levels of procrastination, suggesting that a longer professional career might be associated with a greater capacity for self-regulation.

However, this relationship should be interpreted with caution, as the observed correlation does not imply a causal effect, but rather an association that may be influenced by a set of interrelated factors, such as the development of coping strategies, adaptation to the professional context, or emotional regulation.

Finally, the present study found no significant differences based on the number of children or gender, which supports the relevance of cross-cutting psychological processes over traditional sociodemographic variables. These results align with those reported by Irfan et al. (2024), who found that gender and family type did not influence the level of academic procrastination among future teachers. However, unlike the results obtained in the present study, they did find statistically significant differences based on place of residence (urban or rural areas), with teachers in rural settings scoring higher on academic procrastination.

Importantly, the present study extends previous research by examining these psychological processes in a population of practicing teachers. While much of the existing literature on procrastination and related mechanisms has focused on student samples, teaching represents a professional context with distinct characteristics, including continuous external evaluation, high responsibility, and persistent uncertainty in task outcomes. These features may contribute to the relevance of evaluative concerns and emotional vulnerability processes, making transdiagnostic mechanisms such as intolerance of uncertainty and fear of negative evaluation particularly relevant. Therefore, examining these variables in teachers provides additional insight into how these processes operate in real-world professional settings, beyond academic environments. From a methodological standpoint, the study has significant strengths that reinforce the validity of the results obtained. First, the large sample size allows for stable estimates with sufficient statistical power. Second, the use of a comprehensive analytical approach—including correlational analyses, hierarchical regressions, and a bootstrapped mediation analysis—provides a rigorous and integrated understanding of the relationships among the variables.

However, several limitations should be considered. Given the cross-sectional design of the study, the temporal ordering of the variables cannot be established. Therefore, the observed indirect association between intolerance of uncertainty, fear of negative evaluation, and academic procrastination should not be interpreted as evidence of a causal or directional mechanism. Although the findings are consistent with a mediation model at a statistical level, alternative explanations, including reverse or bidirectional relationships, cannot be ruled out. Future research using longitudinal or experimental designs is necessary to clarify the directionality of these associations. Also, the use of non-probabilistic convenience sampling limits the generalizability of the findings to the broader population of teachers, and the exclusive reliance on self-report measures may introduce potential sources of bias, including social desirability bias, common method variance, and response style effects, which may have influenced the observed associations. Additionally, the absence of longitudinal data prevents examining the temporal directionality and stability of the relationships identified.

Future research should address these limitations by replicating the present study in different educational and professional contexts, employing longitudinal designs, and incorporating multi-method assessment strategies. It would also be valuable to explore the role of additional psychological and contextual variables, such as teacher self-efficacy or institutional support, which may act as mediators or moderators in the relationships examined.

Overall, the present findings suggest that academic procrastination among teachers can be understood as an avoidance-related behavior linked to evaluative concerns that are triggered in contexts of uncertainty. This interpretation contributes to a more integrated understanding of the phenomenon from a transdiagnostic perspective, while highlighting the importance of addressing underlying psychological processes rather than sociodemographic variables or superficial organizational factors. The study provides a relevant empirical basis both for the development of more precise explanatory models and for the design of intervention strategies aimed at reducing procrastination and improving teachers' professional wellbeing.

## 5 Conclusions

The present study examined the role of intolerance of uncertainty and fear of negative evaluation in academic procrastination among secondary school teachers. The findings suggest that fear of negative evaluation represents a key factor associated with procrastination and is linked to the relationship between intolerance of uncertainty and avoidance behavior.

These results contribute to the literature by integrating these constructs within a transdiagnostic framework in a professional context that has received limited attention. From a theoretical perspective, the study supports the importance of understanding procrastination as an emotionally driven process linked to evaluative concerns. From a practical perspective, the findings highlight the relevance of addressing fear of negative evaluation and emotional vulnerability mechanisms in interventions aimed at reducing procrastination and improving teachers' wellbeing.

However, these conclusions should be interpreted in light of the study's limitations, particularly the cross-sectional design, non-probabilistic sampling, and reliance on self-report measures. Future research should extend these findings using longitudinal and multi-method approaches, as well as examining their generalizability across different educational contexts.

## Data Availability

The datasets presented in this article are not readily available because the datasets generated during the current study are available from the corresponding author upon reasonable request. The data are anonymized and subject to ethical and confidentiality restrictions to protect participants' privacy. Requests to access the datasets should be directed to paula.rodriguez@universidadeuropea.es.
